# Supplementary material for: CAP-MAP: cap analysis protocol with minimal analyte processing, a rapid and sensitive approach to analysing mRNA cap structures
Source: Open Biol. 2020 Feb 26;10(2):190306. doi: 10.1098/rsob.190306 (PMC7058934; doi:10.1098/rsob.190306)
Supplement: Supplemental Figures [file rsob190306supp1.pdf]

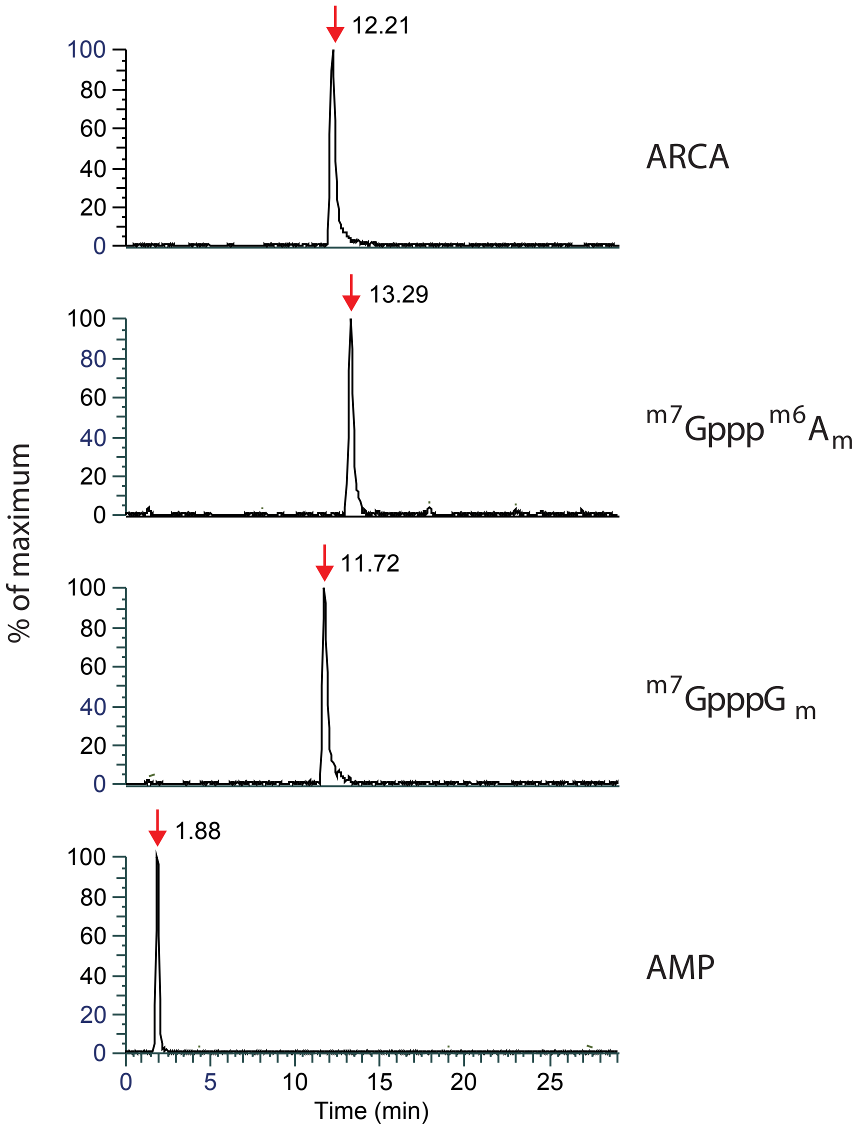

Supplemental figure 1

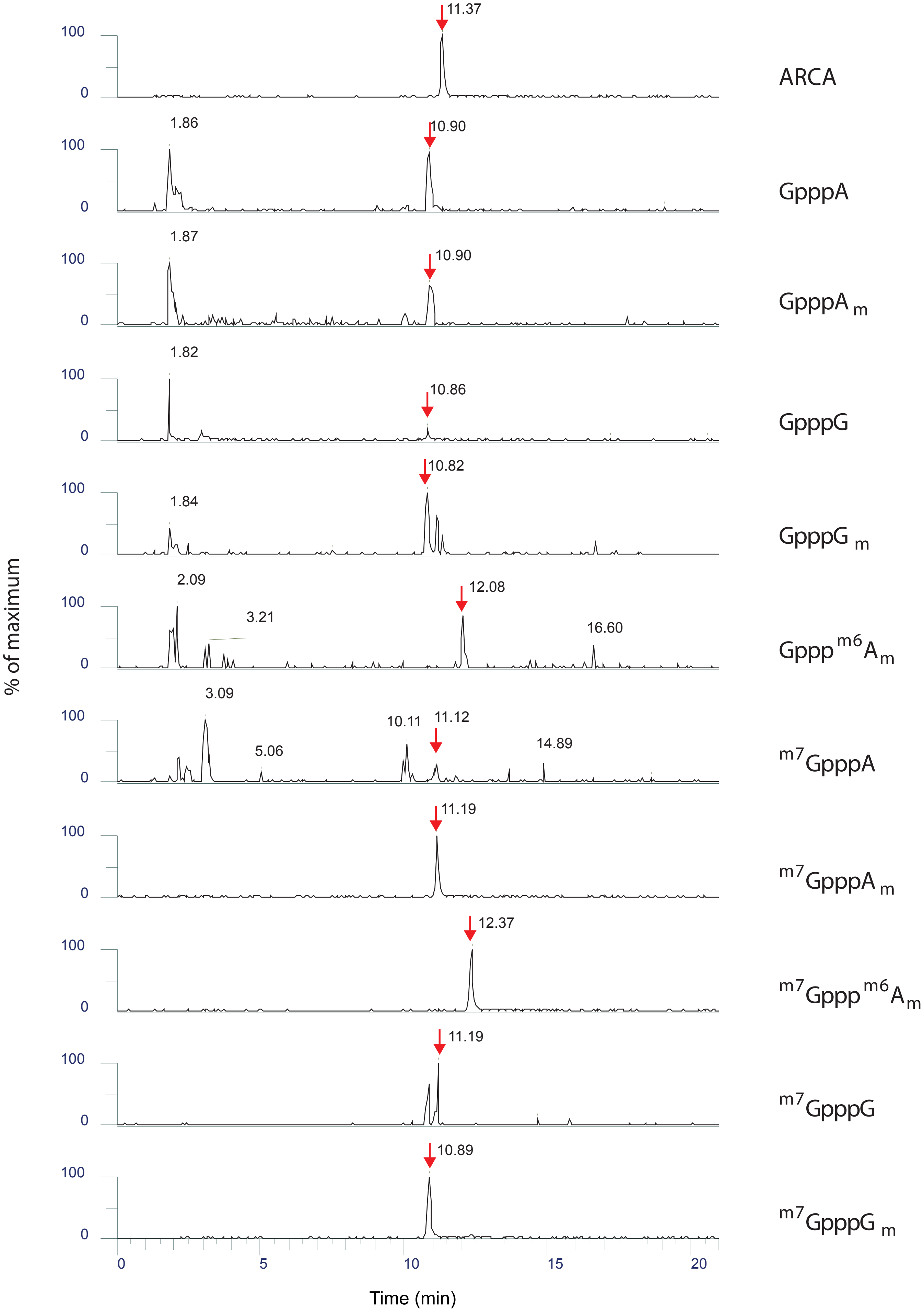

Supplemental figure 2

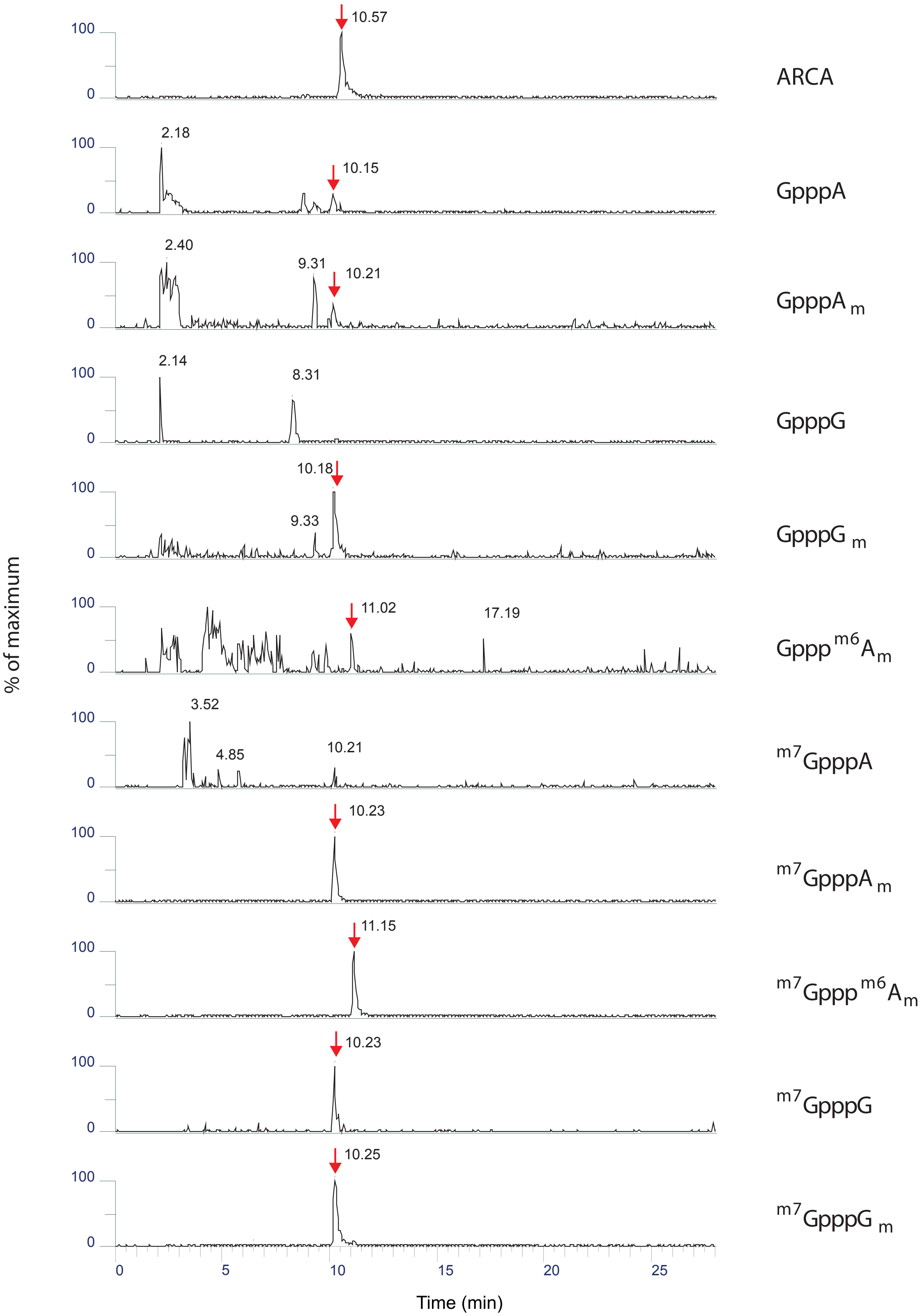

Supplemental figure 3

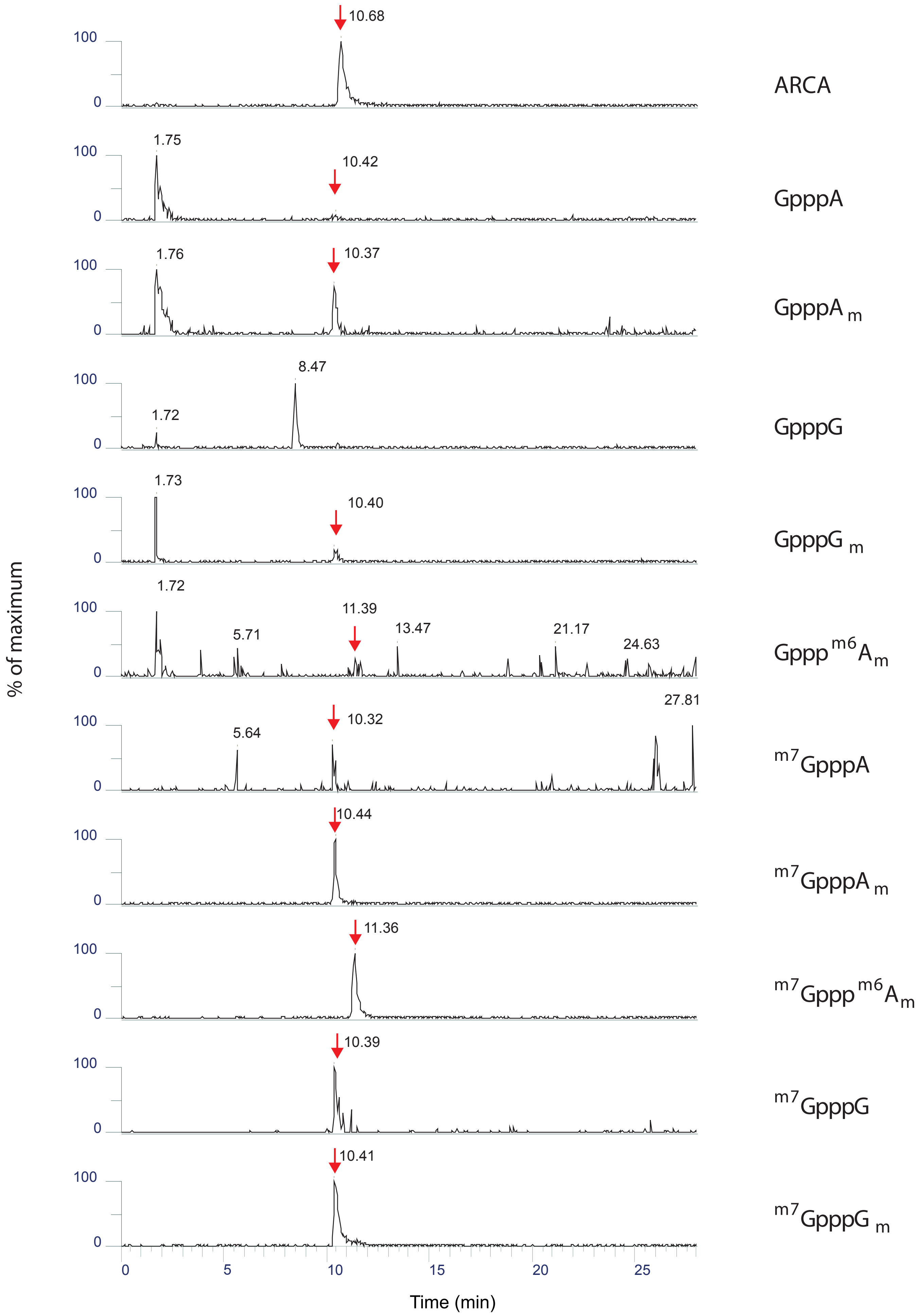

Supplemental figure 4

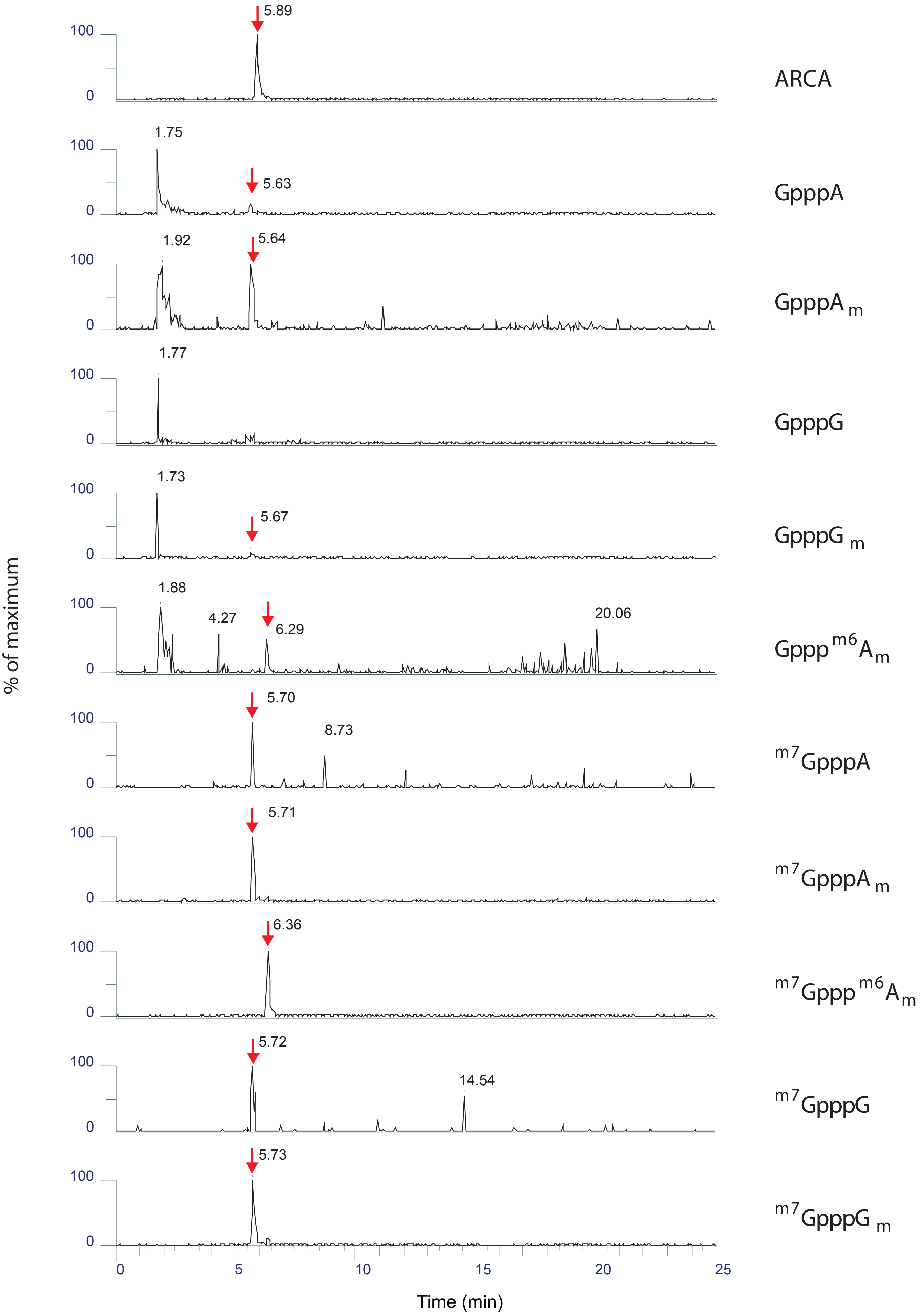

Supplemental figure 5

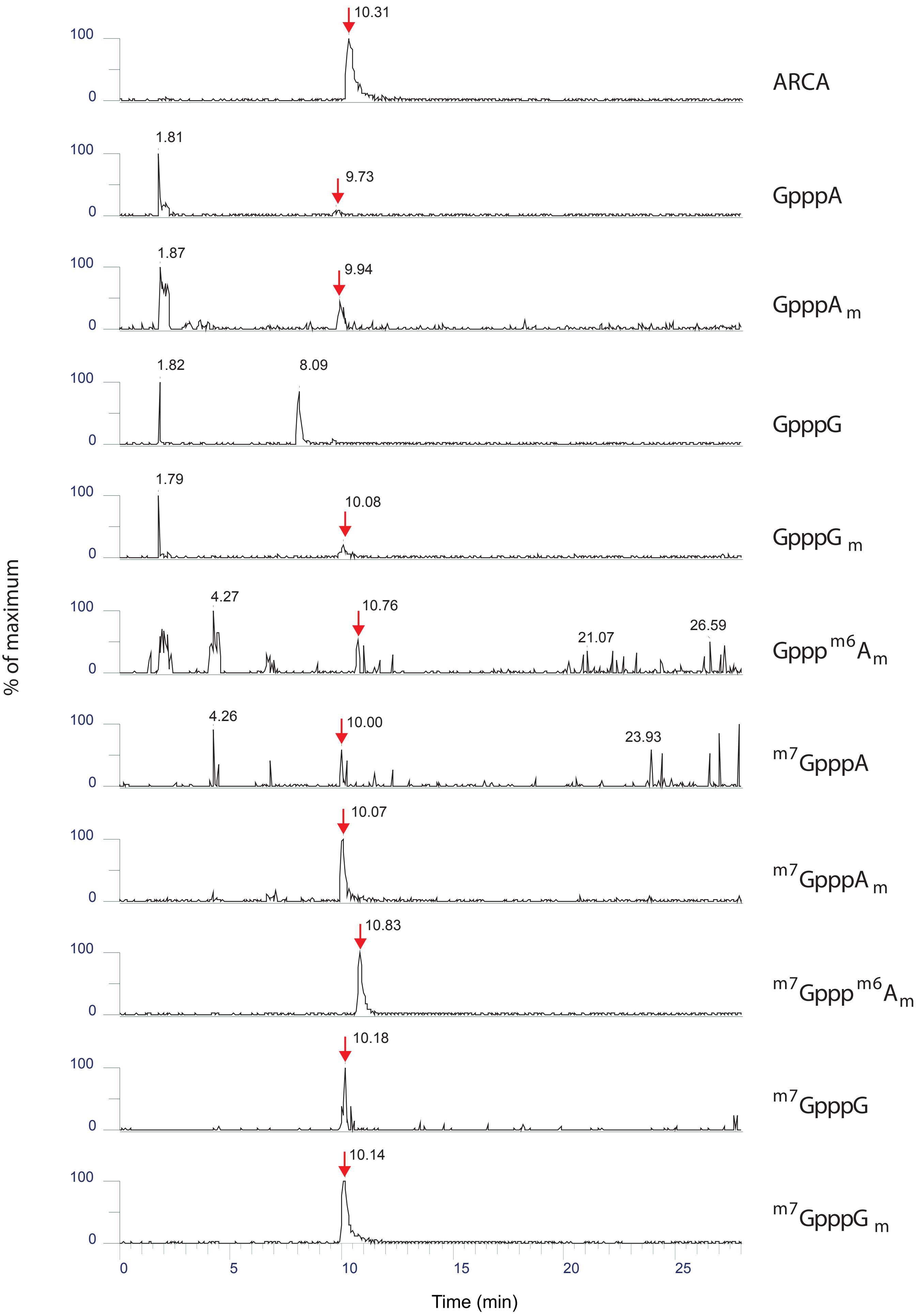

Supplemental figure 6

A

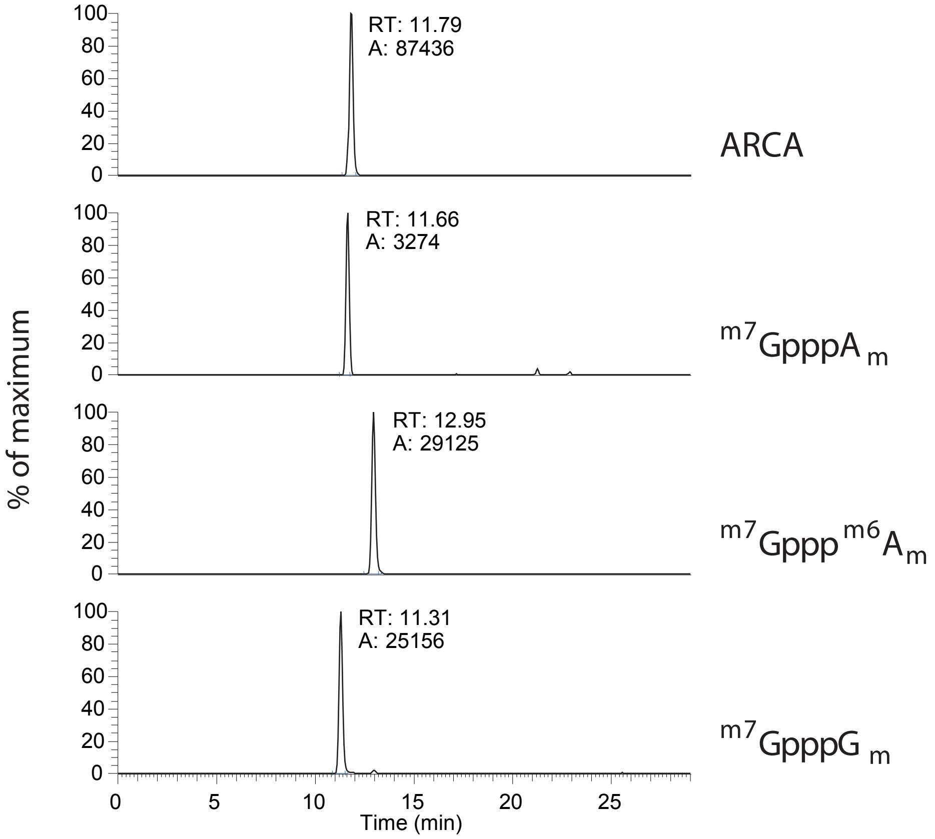

B

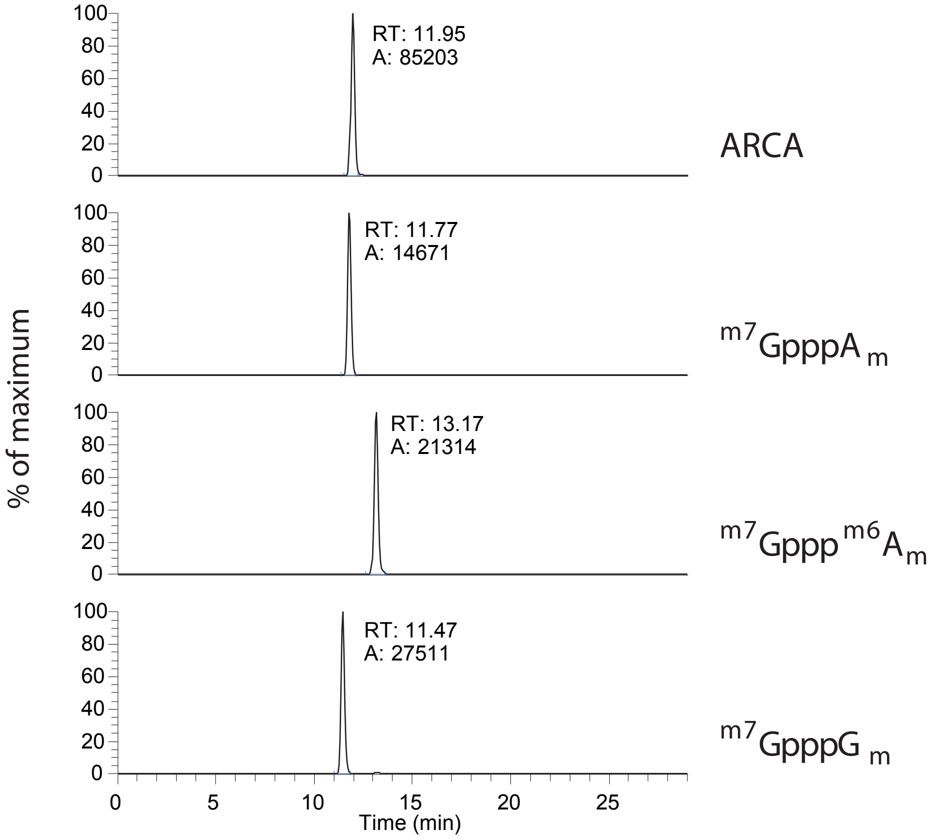

Supplemental figure 7

Supplemental Figure Legends

Supplemental figure 1: Representative chromatogram showing elution of AMP from the column.

AMP, $^{m7}Gppp^{m6}A_{m,}$ $^{m7}GpppG_{m}$, and ARCA were detected in an mRNA sample. Red arrows indicate the peak corresponding to the cap dinucleotide indicated.

Supplemental figure 2: Representative chromatograms from the titration of liver mRNA from figure 5 B and C.

30µg sample shown. Red arrows indicate the peak corresponding to the cap dinucleotide indicated.

Supplemental figure 3: Representative chromatograms from the analysis of liver mRNA from figures 5 A and 6 A, B and C.

Red arrows indicate the peak corresponding to the cap dinucleotide indicated.

Supplemental figure 4: Representative chromatograms from the analysis of CD8 T cell mRNA from figure 6 A, B and C.

Red arrows indicate the peak corresponding to the cap dinucleotide indicated.

Supplemental figure 5: Representative chromatograms from the analysis of heart mRNA from figure 6 A, B and C.

Red arrows indicate the peak corresponding to the cap dinucleotide indicated. These samples were run on a different PGC column to the other samples (which later stopped working), we found that there were column to column differences in the retention times, but the order of elution was maintained.

Supplemental figure 6: Representative chromatograms from the analysis of brain mRNA from figure 6 A, B and C.

Red arrows indicate the peak corresponding to the cap dinucleotide indicated.

Supplemental figure 7: Representative chromatograms from the analysis of HeLa mRNA from figure 7 B and C.

HeLa cells were treated with scrambled siRNA (A) or CAPAM siRNA (B). RT: retention time and A: peak area, are shown.
